# Supplementary material for: Visual processing of musical syntax and its relationship to sight-reading ability
Source: PLoS One. 2026 Mar 11;21(3):e0344490. doi: 10.1371/journal.pone.0344490 (PMC12978459; doi:10.1371/journal.pone.0344490)
Supplement: S1 File — The file contains additional methodological details including participant exclusion criteria, atonalization procedure for creating the atonal sight-reading materials, the complete participant questionnaire, detailed statistical analyses addressing temporal autocorrelation in self-paced reading data, comprehensive documentation of how we handled convergence issues in the mixed-effects models, and robustness analyses examining the consistency of our findings across alternative analytical approaches. (DOCX) [file pone.0344490.s001.docx]

Supplementary Methods and Results

For **Visual processing of musical syntax and its relationship to sight-reading ability**

by Yeoeun Lim and Andrew Goldman

Supplemental methods

Participant exclusion criteria

A total of 23 pianists were recruited for this study. Data from three participants were excluded, resulting in a final sample of 20 participants. The exclusion criteria were as follows: one participant (P001) was excluded because they misinterpreted the instructions and performed the sight-reading task at half the required tempo. A second participant (P003) was excluded because the session was interrupted by an external commitment, requiring them to pause the experiment for an extended period and return later to complete it. A third participant (P009) was excluded for failing to follow task instructions for the self-paced reading task; despite explicit instructions to read each chord carefully, the participant engaged in mechanical clicking through the stimuli, completing the task in less than half the typical time.

Additionally, data from one performance (P015’s atonal piece 2) was excluded from the evaluation of sight-reading performance. The participant did not follow the primary task instruction to maintain tempo with the metronome. The performance contained extensive pauses and note repetitions that caused structural breakdown in the music. This rendered the output of the performance-score alignment algorithm unreliable, thus precluding valid accuracy analysis.

Atonalization procedure

For the sight-reading task, a total of six pieces were used: three tonal pieces selected from the ABRSM collection (Nos. 4, 13, and 15) and three atonal counterparts created by adapting other pieces from the same collection (Nos. 14, 6, and 10).

The atonal versions were created by modifying the pitch content while preserving all other musical elements (e.g., rhythm, meter, articulation, or general texture) from the original pieces. Specifically, chords were altered by shifting selected pitches by a semitone or whole tone, thereby removing functional harmonic relationships (e.g., tonic-dominant movement). In cases where chords contained strong tonal markers (e.g., perfect cadences, leading-tone resolutions, functional bass motion), these were replaced with intervallic structures that avoided tonal attraction. The contour of individual voices was preserved where possible, ensuring similar technical demands across tonal and atonal versions. Additionally, original rhythms were strictly maintained to control for potential confounds related to rhythm processing. The pianistic playability of the atonal versions was carefully considered—chord voicings were adjusted as needed to maintain ergonomic hand positioning, with block chords typically kept within an octave span to match the original versions. This atonalization process resulted in pieces that maintained a similar level of technical difficulty and rhythmic complexity as their tonal counterparts but lacked clear tonal centers or functional harmonic relationships.

Questionnaire

**[1] Background Information**

1. What is your age? ______________________________

2. What is your self-identified gender? _________________________________

3. Which degrees have you completed in piano or collaborative piano? (if any) _____________________________________

4. Which degree program are you enrolled in now? (if any) ___________________________________________________________

5. At what age did you start playing the piano? ________________________ years old

6. At what age did you begin formal piano training (taking regular lessons)? ________________________ years old

7. For how many years have you studied piano at the university level? ________________________ years

8. Please list any other instruments you play besides piano (with years of study for each in parentheses):

**[2] Sight-Reading**

| **Please circle the most appropriate category:** | **1**  **Completely Disagree** | **2**  **Strongly**  **Disagree** | **3**  **Disagree** | **4**  **Neither**  **Agree nor**  **Disagree** | **5**  **Agree** | **6**  **Strongly**  **Agree** | **7**  **Completely**  **Agree** |
| --- | --- | --- | --- | --- | --- | --- | --- |
| 1. I think I am good at sight-reading. | 1 | 2 | 3 | 4 | 5 | 6 | 7 |
| 2. I am a confident sight-reader of classical tonal music (practices and styles like Mozart, Schubert, etc.) | 1 | 2 | 3 | 4 | 5 | 6 | 7 |
| 3. I am a confident sight-reader of atonal music (practices and styles like Schoenberg, Babbitt, etc.) | 1 | 2 | 3 | 4 | 5 | 6 | 7 |

4. How often do you engage in sight-reading? (Choose one)

☐ Every day ☐ 5-6 times per week ☐ 3-4 times per week ☐ 1-2 times per week ☐ 2-3 times per month

☐ Once a month ☐ Several times per year ☐ Rarely or never

5. In what contexts do you typically engage in sight-reading? (Check all that apply)

☐ Solo practice ☐ Accompaniment ☐ Chamber music/Ensemble ☐ Teaching ☐ Auditions/Examinations

☐ Other (please specify): _____________________________________________

**[3] Perceptual Abilities**

| **Please circle the most appropriate category:** | **1**  **Completely Disagree** | **2**  **Strongly**  **Disagree** | **3**  **Disagree** | **4**  **Neither**  **Agree nor**  **Disagree** | **5**  **Agree** | **6**  **Strongly**  **Agree** | **7**  **Completely**  **Agree** |
| --- | --- | --- | --- | --- | --- | --- | --- |
| 1. I am able to judge whether someone is a good singer or not. | 1 | 2 | 3 | 4 | 5 | 6 | 7 |
| 2. I usually know when I’m hearing a song for the first time. | 1 | 2 | 3 | 4 | 5 | 6 | 7 |
| 3. I find it difficult to spot mistakes in a performance of a song even if I know the tune. | 1 | 2 | 3 | 4 | 5 | 6 | 7 |
| 4. I can compare and discuss differences between two performances or versions of the same piece of music. | 1 | 2 | 3 | 4 | 5 | 6 | 7 |
| 5. I have trouble recognizing a familiar song when played in a different way or by a different performer. | 1 | 2 | 3 | 4 | 5 | 6 | 7 |
| 6. I can tell when people sing or play out of time with the beat. | 1 | 2 | 3 | 4 | 5 | 6 | 7 |
| 7. I can tell when people sing or play out of tune. | 1 | 2 | 3 | 4 | 5 | 6 | 7 |
| 8. When I sing, I have no idea whether I’m in tune or not. | 1 | 2 | 3 | 4 | 5 | 6 | 7 |
| 9. When I hear a music I can usually identify its genre. | 1 | 2 | 3 | 4 | 5 | 6 | 7 |

**[4] Musical Training**

| **Please circle the most appropriate category:** | **1**  **Completely Disagree** | **2**  **Strongly**  **Disagree** | **3**  **Disagree** | **4**  **Neither**  **Agree nor**  **Disagree** | **5**  **Agree** | **6**  **Strongly**  **Agree** | **7**  **Completely**  **Agree** |
| --- | --- | --- | --- | --- | --- | --- | --- |
| 1. I have never been complimented for my talents as a musical performer. | 1 | 2 | 3 | 4 | 5 | 6 | 7 |
| 2. I would not consider myself a musician. | 1 | 2 | 3 | 4 | 5 | 6 | 7 |
| **Please circle the most appropriate category** | | | | | | | |
| 3. I engaged in regular, daily practice of a musical instrument (including voice) for _____ years | 0 | 1 | 2 | 3 | 4-5 | 6-9 | 10 or more |
| 4. At the peak of my interest, I practiced _____ hours per day on my primary instrument. | 0 | 0.5 | 1 | 1.5 | 2 | 3-4 | 5 or more |
| 5. I have had formal training in music theory for _____ years. | 0 | 0.5 | 1 | 2 | 3 | 4-6 | 7 or more |
| 6. I have had _____ years of formal training on a musical instrument (including voice) during my lifetime. | 0 | 0.5 | 1 | 2 | 3-5 | 6-9 | 10 or more |
| 7. I can play _____ musical instruments. | 0 | 1 | 2 | 3 | 4 | 5 | 6 or more |

Statistical modeling

This section details the systematic process undertaken to select and validate the final linear mixed-effects model for our study.

Initial and autocorrelation diagnosis

Self-paced reading data present unique statistical challenges due to their sequential nature. Consecutive reading times within a trial often exhibit temporal dependencies, violating the independence assumption of standard regression models. To investigate this in our data, we began with the full dataset of 7,360 (46 progressions × 8 chords × 20 participants). After excluding incorrect responses (14.89% of the original observations), we had 6,264 observations. Outlier identification using all chord positions removed only 12 additional observations (0.19%), resulting in 6,252 observations for initial model exploration. Using this dataset, we fitted a theoretically motivated baseline model (hereafter, Model Simple):

*logRT ~ harmonic_IC + (1 + harmonic_IC | participant)*

This model included harmonic information content (IC) as the primary predictor of interest, with by-participant random intercepts and slopes to account for individual differences in baseline reading speed and sensitivity to harmonic predictability. The random slope for harmonic IC was theoretically motivated by the expectation that participants would vary in their ability to utilize syntactic knowledge during visual processing of chord progressions.

Examination of the residuals from this model revealed substantial positive autocorrelation. The autocorrelation function (ACF) showed a value of 0.18 at lag-1, which exceeded the 95% confidence bounds of ±0.03 (calculated as ±1.96/√6252 for our sample size). This indicates that consecutive residuals were significantly more similar than would be expected by chance. This pattern is typical in self-paced reading studies, where processing spillover effects, motor planning continuity, and attentional fluctuations create dependencies between successive responses. While the fixed effect of harmonic IC was significant (*b* = 2.63, *SE* = 0.40, *t* = 6.55, *p* < .001), the presence of autocorrelation suggested that these estimates might be biased.

Alternative model specifications

To address the autocorrelation issue, we systematically evaluated several alternative model specifications, following the principle of parsimony—using the simplest model that adequately addresses the statistical issues.

**Alternative model 1 (nested structure; hereafter, Model A1)**

Our first attempt involved adding crossed random effects for the hierarchical structure of the stimuli:

*logRT ~ harmonic_IC + (1 + harmonic_IC | participant) + (1 | progression/measure)*

This specification acknowledged that chords (measures) are nested within chord progressions, which might create dependencies. The model converged without warnings, and the additional random effects captured some variance (progression: Var = 0.02; measure within progression: Var = 0.01). However, this model failed to reduce the autocorrelation, with the ACF at lag-1 remaining at 0.19, still well above the 95% confidence bounds. The fixed effect of harmonic IC was somewhat attenuated (*b* = 2.10, *SE* = 0.76, *t* = 2.77, *p* = .006) but remained significant. Model comparison using likelihood ratio tests showed that while Model A1 had a significantly better fit than Model Simple (χ²(2) = 287.40, *p* < .001), the persistence of autocorrelation suggested that the temporal dependencies in reading times were not adequately captured by the hierarchical structure of items alone.

**Alternative model 2 (autoregressive with lag-1; hereafter, Model A2)**

Following recommendations from the psycholinguistic literature [1], we next included the lag-1 reading time as fixed effect:

*logRT ~ lag1_RT + harmonic_IC + (1 + harmonic_IC | participant)*

This approach directly models the temporal dependency by controlling for the previous reading time. The lag-1 predictor was highly significant (*b* = 0.26, *SE* = 0.01, *t* = 18.87, *p* < .001), confirming the presence of temporal dependencies. The inclusion of lag-1 RT substantially reduced the primary autocorrelation from 0.18 to -0.09, with the ACF now falling below the adjusted 95% confidence bounds of ±0.03 (adjusted for the reduced sample size of 5,469 observations after removing first observations). The negative value at lag-1 suggests a slight overcorrection, a state generally considered preferable to uncorrected positive autocorrelation, which is known to inflate Type I error rates. Importantly, the effect of harmonic IC remained highly significant (*b* = 1.70, *SE* = 0.32, *t* = 5.40, *p* < .001), though its magnitude was reduced compared to Model Simple, reflecting a more conservative estimate after controlling for temporal dependencies. The model showed excellent convergence properties with no warnings, and the random effects maintained reasonable variance estimates (intercept: Var = 0.08; harmonic IC slope: Var = 0.04).

Despite the substantial improvement in primary autocorrelation, some temporal dependencies remained at higher lags, particularly at lag-2 (ACF = 0.13) and lag-7 (ACF = 0.10). However, simulation studies in the mixed-effects modeling literature have demonstrated that moderate levels of remaining autocorrelation have minimal impact on fixed effect estimates, particularly when the primary lag-1 autocorrelation is controlled [2]. To further evaluate the robustness of our findings, we conducted a sensitivity analysis: even if the standard error were doubled to account for potential underestimation—a highly conservative correction—the *t*-value would be 2.70, maintaining significance at *p* = .007.

**Alternative model 3 (ar(2) structure; hereafter, Model A3)**

Given that some autocorrelation remained at lag-2 (ACF = 0.13, exceeding the 95% bounds), we attempted a second-order autoregressive model:

*logRT ~ lag1_RT + lag2_RT + harmonic_IC + (1 + harmonic_IC | participant)*

While the lag-2 predictor showed some significance (*b* = 0.09, *SE* = 0.01, *t* = 6.21, *p* < .001), this model resulted in a singular fit warning, indicating that the random effects structure could not be reliably estimated with the additional complexity. The variance component for the random slope of harmonic IC approached zero, and the optimization algorithm reported convergence issues. This instability suggested that the Model A3 was overparameterized for our data structure, making Model A2 the most viable option.

Final model selection

Based on this systematic evaluation, we selected Model A2 with lag-1 RT as a covariate for all subsequent analyses. This decision was justified by: (1) substantial reduction in autocorrelation to acceptable levels, (2) model stability and convergence, (3) theoretical interpretability, and (4) consistency with established practices in psycholinguistic research. The inclusion of lag-1 RT necessitated excluding first-chord observations from all analyses, which is why the Results section in the main manuscript reports descriptive statistics for the self-paced reading task and mixed models based on chords 2–8 only.

Supplemental results

Handling convergence issues in mixed models

Mixed-effects models can encounter convergence issues when the optimization algorithms have difficulty finding parameter estimates or when the random effects structure is overparameterized relative to the available data. In our analyses, particularly for the interaction models, we encountered different types of convergence issues across conditions and performance metrics, which we addressed using established best practices from the mixed modelling literature [3,4]. This section provides a comprehensive account of these issues and their resolutions.

Two-way interaction models

**bn-MAD in the tonal sight-reading condition**

The two-way interaction model for bn-MAD in the tonal sight-reading condition initially produced a convergence warning:

*lmer(logRT ~ logRT_lag1_c +*

*harmonic_IC_c * bn-MAD_c +*

*(1 + harmonic_IC_c | participant),*

*data = df_item_tonal)*

*Warning message: In checkConv(attr(opt, “derivs”), opt$par, ctrl = control$checkConv, : Model failed to converge

with max|grad| = 0.00305214 (tol = 0.002, component 1)

This warning indicates that the gradient value (0.00305214) exceeded the default tolerance threshold (0.002), suggesting that the optimization algorithm had difficulty finding the precise minimum of the likelihood surface. Such convergence warnings are common in complex mixed models and do not necessarily indicate model misspecification [3]. Following recommendations in the lme4 documentation and the package vignette on performance optimization, we changed the optimizer from the default “nloptwrap” to “bobyqa” (Bound Optimization BY Quadratic Approximation) [5]:

*lmer(logRT ~ logRT_lag1_c +*

*harmonic_IC_c * bn-MAD_c +*

*(1 + harmonic_IC_c | participant),*

*data = df_item_tonal,*

*control = lmerControl(optimizer = “bobyqa”))*

This change successfully resolved the convergence warning without substantively affecting the model estimates. The use of alternative optimizers is a well-established approach for addressing convergence problems in mixed-effects models [4] (see also the GLMM FAQ maintained by Ben Bolker). Importantly, diagnostic testing confirmed that multiple optimizers produced identical parameter estimates, indicating that the original warning reflected numerical optimization challenges rather than fundamental model problems.

**CV in the atonal sight-reading condition**

The two-way interaction model for *CV* in the atonal sight-reading condition resulted in a singular fit:

*lmer(logRT ~ logRT_lag1_c +*

*harmonic_IC_c * CV_c +*

*(1 + harmonic_IC_c | participant),*

*data = df_item_atonal)*

*boundary (singular) fit: see help(‘isSingular’)

*Random effects:

Groups Name Variance Std.Dev. Corr

participant (Intercept) 0.07025 0.2650

harmonicIC_c 0.01158 0.1075 -1.00

The perfect negative correlation (-1.00) between random intercepts and slopes, combined with the extremely small random slope variance (0.01158), indicates that the random effects structure was overparameterized relative to the variation present in the data [3]. This is a well-documented phenomenon in mixed modeling, occurring when the model attempts to estimate more variance components than the data can support [6].

Following the principle of building parsimonious models that the data can support [3], we systematically tested alternative specifications. Neither alternative optimizers (bobyqa, Nelder-Mead, nlminbwrap variants) nor the removal of correlation between random effects (|| notation) successfully resolved the singularity, indicating that the issue was fundamental rather than numerical. Therefore, we adopted a random intercept-only model:

*lmer(logRT ~ logRT_lag1_c +*

*harmonic_IC_c * CV_c +*

*(1 | participant),*

*data = df_item_atonal)*

This simplification follows recommendations in the mixed modeling literature for handling singular fits [7,8]. Importantly, comparison of fixed effects between the original and simplified models showed minimal changes (maximum 0.28%), confirming that our substantive conclusions are robust to the random effects specification.

Three-way interaction models

**bn-MAD in the atonal sight-reading condition**

The three-way interaction model for bn-MAD in the atonal sight-reading condition encountered convergence issues:

*lmer(logRT ~ logRT_lag1_c +*

*harmonic_IC_c * bn-MAD_c * NESI_score_c +*

*(1 + harmonic_IC_c | participant),*

*data = df_item_atonal)*

*Warning message:

Model failed to converge with 1 negative eigenvalue: -9.0e-01

boundary (singular) fit: see help(‘isSingular’)

*Random effects:

Groups Name Variance Std.Dev. Corr

participant (Intercept) 0.08104 0.2847

harmonicIC_c 0.04195 0.2048 -1.00

The negative eigenvalue warning, combined with perfect negative correlation between random effects, indicates serious convergence problems. However, unlike the two-way interaction model for *CV* in the atonal sight-reading condition, the random slope variance here remained substantial (0.04195), suggesting meaningful between-participant variation that should be retained if possible. Following recommendations for handling convergence issues while maintaining model complexity [7] (afex package documentation), we removed the correlation between random effects using the || notation:

*lmer(logRT ~ logRT_lag1_c +*

*harmonic_IC_c * bn-MAD_c * NESI_score_c +*

*(1 + harmonic_IC_c || participant),*

*data = df_item_atonal)*

This specification maintains both random intercepts and slopes while constraining their correlation to zero, effectively reducing the number of parameters to be estimated. This approach is recommended when the full random effects structure is theoretically motivated but computationally problematic [6]. The model converged successfully without warnings, and the random slope standard deviation remained meaningful (SD = 0.2048), supporting our decision to retain the random slopes.

**CV in the tonal sight-reading condition**

The three-way interaction model for *CV* in the tonal sight-reading condition also resulted in a singular fit:

*lmer(logRT ~ logRT_lag1_c +*

*harmonic_IC_c * CV_c * NESI_score_c +*

*(1 + harmonic_IC_c | participant),*

*data = df_item_tonal)*

*boundary (singular) fit: see help(‘isSingular’)

*Random effects:

Groups Name Variance Std.Dev. Corr

participant (Intercept) 0.08190 0.2862

harmonicIC_c 0.04044 0.2011 -1.00

As with the three-way interaction model for bn-MAD in the atonal condition, the perfect negative correlation indicated overparameterization. Despite the apparently reasonable random slope variance, comprehensive testing revealed that the singularity persisted across all attempted solutions (alternative optimizers and || notation). This pattern suggests that the *CV* metric captures less between-participant heterogeneity in syntactic processing effects than the bn-MAD metric. Following the principle of model parsimony and the recommendations for handling persistent singular fits [3,6], we simplified to a random intercept-only structure:

*lmer(logRT ~ logRT_lag1_c +*

*harmonic_IC_c * CV_c * NESI_score_c +*

*(1 | participant),*

*data = df_item_tonal)*

While this represents a substantial simplification, it is preferable to retaining a singular model that may produce unreliable estimates [8]. The fixed effects showed acceptable stability (maximum change 7.62%), with primary effects maintaining their significance.

Summary and implications

The model adjustments described above represent standard practice in mixed-effects modeling and follow established guidelines for addressing convergence issues [3,6,7]. These adjustments reflect a principled approach to model specification that balances theoretical considerations with the practical constraints imposed by the data.

For bn-MAD models, the data generally supported more complex random effects structures. In the tonal sight-reading condition, simple optimizer changes sufficed to achieve convergence, preserving the full theoretical model. In the atonal sight-reading condition’s three-way interaction model, removing the correlation between random effects (|| notation) provided a solution that maintained both random components while addressing the computational issues. This pattern suggests that bn-MAD captures meaningful individual differences in how timing precision moderates harmonic syntactic processing.

In contrast, *CV* models consistently required simplification to random intercept-only structures. The minimal random slope variances and persistent singular fits across sight-reading conditions indicate that participants showed remarkably uniform patterns when timing consistency was measured through *CV*. This systematic difference between metrics is theoretically informative: absolute timing deviations (bn-MAD) appear to capture more heterogeneous aspects of sight-reading proficiency than relative consistency measures (*CV*).

Critically, the stability of fixed effects across model specifications supports the robustness of our substantive conclusions. When only optimizers were changed, fixed effects remained virtually identical. Even when structural simplifications were necessary, changes in fixed effects remained within acceptable ranges, and the pattern of significant results was preserved. This robustness is consistent with simulation studies showing that fixed effects estimates in mixed models are generally robust to random effects misspecification [9].

The systematic patterns in our convergence issues—varying by metric but consistent across conditions—suggest that they reflect genuine properties of how different aspects of sight-reading proficiency relate to syntactic processing, rather than mere statistical artifacts. These findings contribute to the growing understanding of how to handle convergence issues in mixed models while maintaining scientific rigor and interpretability.

Robustness analyses for the harmonic IC effect on reading times

The main analysis revealed that harmonically less predictable chords (higher harmonic IC) were associated with longer reading times, consistent with the interpretation that participants processed harmonic syntax while silently reading chord notation. However, there is a possibility that this relationship might potentially be confounded by other stimulus characteristics. Here we report a series of robustness analyses examining two classes of potential confounds: visual complexity as indexed by accidental count (the number of sharps, flats, and naturals) and chord spacing (bass to soprano in semitones) and progression-final position (“wrap-up”) effects.

To ensure consistency between the stimulus-level correlation analyses reported here and the linear mixed-effects models, all analyses of stimulus characteristics were conducted on chord positions 2–8 (*n* = 322 chords; 46 progressions × 7 positions). Position 1 was excluded because the linear mixed-effects models included a lag-1 reading time predictor to control for temporal autocorrelation, which necessarily removes the first chord of each progression from the behavioral analyses. By applying the same constraint to our correlation analyses, we ensured that the stimulus characteristics examined here directly correspond to the data included in the confirmatory models.

Visual complexity

Musical notation varies in visual complexity, and more complex visual displays may require longer processing times independent of their harmonic content. We considered two aspects of visual complexity that could plausibly covary with harmonic predictability. First, harmonically less expected chords in tonal music often involve chromatic alterations, which manifest in notation as accidentals (e.g., sharps, flats, or naturals). A chord containing multiple accidentals presents a denser visual array that may take longer to decode. Second, chord voicing affects the vertical spread of notes on the staff: widely spaced chords may require the reader to scan a larger visual area, potentially increasing reading times. If either of these visual factors were strongly associated with harmonic IC, the apparent IC effect on reading times might partly or wholly reflect perceptual processing demands rather than harmonic-syntactic processing.

**Descriptive statistics**

For chord positions 2–8 (*n* = 322), harmonic IC values ranged from 0.23 to 0.49 (*M* = 0.34, *SD* = 0.04). Accidental count ranged from 0 to 3 per chord (*M* = 0.44, *SD* = 0.76). The majority of chords contained no accidentals (225 of 322 chords; 69.9%), while 62 chords (19.3%) contained one accidental, 26 chords (8.1%) contained two, and 9 chords (2.8%) contained three. Chord spacing, defined as the interval from the bass to the soprano voice in semitones, ranged from 7 to 40 semitones (*M* = 23.03, *SD* = 6.26).

**Correlational analyses**

We used Spearman correlations due to the non-normal distributions of all variables (Shapiro-Wilk tests: harmonic IC, *W* = 0.94, *p* < .001; accidentals, *W* = 0.62, *p* < .001; spacing: *W* = 0.98, *p* < .001). Neither accidental count (Spearman rho = .02, *p* = .731) nor chord spacing (Spearman rho = .08, *p* = .166) was significantly associated with harmonic IC. This suggests that in our stimulus set, harmonically less predictable chords were not systematically more visually complex, alleviating concerns about collinearity between these variables. Additionally, the two visual complexity measures were not significantly correlated with each other (Spearman rho = .04, *p* = .427), suggesting they capture distinct aspects of visual complexity. Chord position showed a moderate negative correlation with harmonic IC (Spearman rho = -.36, *p* < .001), a pattern examined in the subsequent final position analyses.

To further confirm the independence of visual complexity and harmonic predictability, we compared IC values between chords with and without accidentals. Chords with zero accidentals (*n* = 225, *M* = 0.33, *SD* = 0.03) did not differ significantly in harmonic IC from chords with one or more accidentals (*n* = 97, *M* = 0.34, *SD* = 0.03; Mann-Whitney *U* = 10760.5, *p* = .843, *r* = .01). Similarly, a Kruskal-Wallis test comparing harmonic IC across spacing tertiles (narrow: *M* = 16.2 semitones; medium: *M* = 23.5 semitones; wide: *M* = 30.5 semitones) revealed no significant differences in harmonic IC (*H* = 4.12, *df* = 2, *p* = .127, *η²* = .01).

**Linear mixed-effects models with visual complexity covariates**

Given that the visual complexity measures examined here were not significantly correlated with harmonic IC, we nonetheless tested whether controlling for visual complexity would alter the harmonic IC effect on reading times (see S1 Table 1 for full results). When accidental count was added as a covariate, accidentals significantly predicted reading times (*p* < .001), with chords containing more accidentals eliciting longer reading times. Importantly, the harmonic IC effect remained significant (*p* < .001), with a 13.7% reduction in the coefficient compared to the original model.

When chord spacing was added as a covariate, spacing did not significantly predict reading times (*p* = .453), suggesting that the vertical extent of chords on the staff does not independently influence processing time. The harmonic IC effect remained virtually unchanged (*p* < .001; 1.7% reduction from the original model).

As a more stringent test, we restricted the analysis to chords with zero accidentals, thereby eliminating any potential influence of accidentals on reading times. Despite the reduced sample size (*n* = 3,874 observations, 70.8% of the original data), the IC effect remained significant (*p* = .002). This model showed a singular fit warning due to a perfect negative correlation between random intercepts and slopes, which can occur when the data do not support the full random effects structure; however, fixed effects estimates typically remain unbiased under such conditions [7].

Collectively, these analyses provide evidence against the possibility that visual complexity—at least as indexed by accidental count and chord spacing—accounts for the observed harmonic IC effect.

**S1 Table 1. Linear Mixed-Effects Models Testing Robustness of the Harmonic IC Effect on Reading Times.**

| **Model** | **n** | **b** | **SE** | **df** | **t** | **p** | **95% CI** | **Δ** |
| --- | --- | --- | --- | --- | --- | --- | --- | --- |
| Original | 5,474 | 1.75 | 0.31 | 21.53 | 5.58 | < .001 | [1.13, 2.36] | — |
| + Accidentals | 5,474 | 1.51 | 0.31 | 21.69 | 4.86 | < .001 | [0.90, 2.12] | -13.7% |
| + Spacing | 5,474 | 1.72 | 0.32 | 23.50 | 5.44 | < .001 | [1.10, 2.34] | -1.7% |
| Excl. position 8 | 4,692 | 1.18 | 0.35 | 320.93 | 3.40 | < .001 | [0.50, 1.87] | -32.3% |
| Accidentals = 0 only | 3,874 | 1.10 | 0.36 | 284.39 | 3.06 | .002 | [0.39, 1.81] | -37.0% |

Abbreviations: Harmonic IC = harmonic information content; n = number of observations; b = unstandardized regression coefficient for harmonic IC; SE = standard error; df = Satterthwaite degrees of freedom; p = *p*-value; CI = confidence interval; Δ = percentage change in coefficient relative to the original model.

Notes:

Original = model with harmonic IC as the predictor of interest (no additional covariates beyond lag-1 reading time); + Accidentals = original model with accidental count added as a covariate; + Spacing = original model with chord spacing (bass-to-soprano interval in semitones) added as a covariate; Excl. position 8 = original model excluding chords at position 8 (progression-final position); Accidentals = 0 only = original model restricted to chords without accidentals. The dependent variable was log-transformed reading time. All models included lag-1 log reading time as a covariate and participant as a random effect with random intercepts and random slopes for harmonic IC. Position 1 was excluded from all models due to the lag-1 predictor. The “Accidentals = 0 only” model produced a singular fit warning; fixed effects estimates remain interpretable but should be considered with caution [7]. Confidence intervals were computed using the Wald method.

Progression-final position and wrap-up effects

In language comprehension, readers tend to slow down at the ends of sentences or clauses to integrate the preceding information—a phenomenon known as wrap-up effects [10]. An analogous process might occur in music reading, with participants pausing at the ends of chord progressions to consolidate harmonic information. If high-IC chords were disproportionately located at final positions, the observed correlation between harmonic IC and reading times might partly reflect such end-of-sequence processes rather than chord-by-chord syntactic processing. To evaluate this possibility, we first examined the distribution of harmonic IC across chord positions and then conducted a sensitivity analysis excluding progression-final chords.

**The distribution of harmonic IC across positions**

S1 Table 2 presents harmonic IC values by chord position. As noted above, position 1 was excluded from all behavioral analyses because the lag-1 reading time predictor removes first-position observations.

More critical for evaluating wrap-up effects is harmonic IC at final positions. Position 8 (the final chord) had the lowest mean harmonic IC (*M* = 0.32, *SD* = 0.03) among positions 2–8. A Mann-Whitney U test confirmed that position 8 had significantly lower harmonic IC than positions 2–7 combined (*M* = 0.34, *SD* = 0.03; *U* = 4510, *p* = .002, *r* = .18). This pattern is also reflected in the moderate negative correlation between chord position and harmonic IC reported above. This distribution has important implications for interpreting the IC effect we observed. Because final-position chords have the lowest (not highest) harmonic IC values, any wrap-up-related slowing at these positions would actually work against finding an IC effect. That is, if participants lingered at position 8 regardless of harmonic IC, and position 8 chords happened to be the most predictable, this would attenuate rather than inflate the positive relationship between harmonic IC and reading times. The observed IC effect is therefore likely a conservative estimate of the true effect.

**S1 Table 2. Harmonic IC by Chord Position.**

| **Position** | **n** | **M** | **SD** | **Median** |
| --- | --- | --- | --- | --- |
| 2 | 46 | 0.35 | 0.02 | 0.35 |
| 3 | 46 | 0.35 | 0.01 | 0.35 |
| 4 | 46 | 0.33 | 0.04 | 0.34 |
| 5 | 46 | 0.33 | 0.03 | 0.34 |
| 6 | 46 | 0.32 | 0.04 | 0.33 |
| 7 | 46 | 0.33 | 0.04 | 0.33 |
| 8 | 46 | 0.32 | 0.03 | 0.33 |

Abbreviations: Harmonic IC = harmonic information content; n = number of observations; M = mean; SD = standard deviation.

Notes:

Position 1 was excluded from all behavioral analyses because the lag-1 reading time predictor necessarily removes first-position observations. Statistics for chord positions 2–8 are based on the data included in the linear mixed-effects models.

**Sensitivity analysis excluding progression-final position**

Despite the favorable distribution of harmonic IC across positions, we conducted a sensitivity analysis to directly test whether the IC effect depends on progression-final observations. The analysis excluded all chords at position 8 (*n* = 4,692 observations remaining). The harmonic IC effect remained significant (*p* < .001; see S1 Table 1 for coefficient estimates and confidence intervals). The coefficient was reduced by 32.3% compared to the original model, which is expected given the removal of approximately one-seventh of the data and the associated reduction in statistical power. The persistence of a significant effect provides evidence against the possibility that the IC-RT relationship is primarily driven by end-of-sequence processing.

Summary of the robustness analyses

Across all robustness analyses, the harmonic IC effect on reading times remained statistically significant. The effect was robust to controlling for accidental count, controlling for chord spacing, restricting the analysis to chords without accidentals, and excluding final-position chords. These findings strengthen the interpretation that our participants were sensitive to harmonic syntax when silently reading chord progressions.

References

1. Baayen RH, Milin P. Analyzing reaction times. Int J Psychol Res. 2010;3: 12-28. doi: 10.21500/20112084.807.

2. Baayen RH, Vasishth S, Kliegl R, Bates D. The cave of shadows: Addressing the human factor with generalized additive mixed models. J Mem Lang. 2017;94: 206-234. doi: 10.1016/j.jml.2016.11.006.

3. Bates D, Mächler M, Bolker B, Walker S. Fitting linear mixed-effects models using lme4. J Stat Softw. 2015;67: 1-48. doi: 10.18637/jss.v067.i01.

4. Bolker BM, Brooks ME, Clark CJ, Geange SW, Poulsen JR, Stevens MHH, et al. Generalized linear mixed models: a practical guide for ecology and evolution. Trends Ecol Evol. 2009;24: 127-135. doi: 10.1016/j.tree.2008.10.008.

5. Powell MJ. The BOBYQA algorithm for bound constrained optimization without derivatives. Cambridge NA Report NA2009/06. Cambridge: University of Cambridge; 2009.

6. Matuschek H, Kliegl R, Vasishth S, Baayen H, Bates D. Balancing Type I error and power in linear mixed models. J Mem Lang. 2017;94: 305-315. doi: 10.1016/j.jml.2017.01.001.

7. Barr DJ, Levy R, Scheepers C, Tily HJ. Random effects structure for confirmatory hypothesis testing: Keep it maximal. J Mem Lang. 2013;68: 255-278. doi: 10.1016/j.jml.2012.11.001.

8. Singmann H, Kellen D. An introduction to mixed models for experimental psychology. In: Spieler DH, Schumacher E, editors. New methods in cognitive psychology. New York: Psychology Press; 2019. pp. 4-31.

9. Schielzeth H, Forstmeier W. Conclusions beyond support: Overconfident estimates in mixed models. Behav Ecol. 2009;20: 416-420. doi: 10.1093/beheco/arn145.

10. Just MA, Carpenter PA. A theory of reading: From eye fixations to comprehension. Psychol Rev. 1980;87: 329-354. doi: 10.1037/0033-295x.87.4.329.
