# Supplementary material for: Visual processing of musical syntax and its relationship to sight-reading ability
Source: PLoS One. 2026 Mar 11;21(3):e0344490. doi: 10.1371/journal.pone.0344490 (PMC12978459; doi:10.1371/journal.pone.0344490)
Supplement: Attachment — Submitted filename: Response to Reviewers.pdf [file pone.0344490.s003.pdf]

Response to Reviewers

1. Comments from Editor

1.1. For the Manuscript:

The external reviews (appended below) identify significant strengths in your experimental design but raise substantial concerns regarding your theoretical framing and computational modeling. Additionally, during my own assessment of the manuscript, I identified a critical methodological discrepancy regarding statistical controls for visual complexity that was not explicitly detailed by the reviewers but must be resolved.

Below, I have separated my own comments from those provided by the external reviewers.

I observe a discrepancy between your Discussion and your Results sections regarding visual complexity. In the Discussion, you explicitly claim that "harmonic IC—not visual complexity such as the number of accidentals or note density—predicted RTs". However, upon reviewing your Methods section and the model results in Table 3, I found no indication that visual complexity (e.g., number of accidentals, note count, or pixel density) was included as a covariate in your linear mixed-effects models.

In Western tonal notation, "unpredictable" chords often involve chromaticism, which results in a higher density of accidentals. Prior research in music reading has consistently demonstrated that visual features such as note density and accidentals significantly impact processing speed and eye movements (e.g., *Halpern & Bower, 1982*; *K Kopiez & Lee, 2008*). Without explicitly controlling for visual density in your model, I cannot accept the claim that longer Reading Times (RT) are purely due to syntactic processing; they may simply reflect the perceptual burden of decoding more visual symbols. You must resolve this by either adding these covariates to your model or revising your claims to reflect this confound.

Response: We are grateful to the Editor for identifying this important gap in our original analysis. The concern that visual complexity might confound the IC-RT relationship is well-founded, and we appreciate the opportunity to address it thoroughly.

Following the Editor's guidance, we operationalized visual complexity using two measures: 1) accidental count, defined as the number of sharps, flats, and naturals per chord (range: 0–3 accidentals in our stimulus set), and 2) chord spacing, defined as the intervallic distance from the bass to the soprano voice in semitones (range: 7–40 semitones in our stimulus set). We selected these measures because accidentals can increase visual density, while wider spacing may require the reader to scan a larger vertical area on the staff—both of which could plausibly increase reading times independent of harmonic content.

To ensure consistency between these stimulus-level analyses and our linear mixed-effects models, all correlational analyses were conducted on chord positions 2–8 (n = 322 chords; 46 progressions × 7 positions). Position 1 was excluded because the linear mixed-effects models included a lag-1 reading time predictor to control for temporal autocorrelation, which necessarily

removes the first chord of each progression from the behavioral analyses. By applying the same constraint to our correlation analyses, we ensured that the stimulus characteristics examined correspond directly to the data included in the confirmatory models.

An initial correlational analysis revealed that neither measure was significantly associated with harmonic IC in our stimulus set (accidentals: Spearman rho = .02, $p = .731$; spacing: Spearman rho = .08, $p = .166$; $n = 322$ chords). This lack of significant association suggests that, at least for these two indices, harmonically less predictable chords were not significantly more visually complex in the materials used here.

Nevertheless, we conducted a series of robustness analyses to directly test whether visual complexity could account for the IC effect. When accidental count was added as a covariate in the linear mixed-effects model, the effect of harmonic IC remained significant ($b = 1.51$, $SE = 0.31$, $t(21.69) = 4.86$, $p < .001$), with a 13.7% reduction in the coefficient relative to the original model. Similarly, when chord spacing was included as a covariate, the IC effect remained significant with minimal change in the coefficient ($b = 1.72$, $SE = 0.32$, $t(23.50) = 5.44$, $p < .001$; 1.7% reduction). Notably, chord spacing itself did not significantly predict reading times ($p = .453$), suggesting that the vertical extent of chords on the staff may not independently influence processing time.

As a more stringent test, we restricted the analysis to chords containing no accidentals ($n = 3{,}874$ observations; 70.8% of the data), thereby holding accidental-related visual complexity constant. In this visually homogenous subset, the IC effect remained significant ($b = 1.10$, $SE = 0.36$, $t(284.39) = 3.06$, $p = .002$).

We have now incorporated these analyses into the Results section (pp.43–45) and provided full details in S1 File (pp.17–20). We have also revised the relevant passage in the Discussion (pp.52–53) to accurately reflect these findings. We believe these additions strengthen the manuscript by providing evidence that the observed reading time differences depending on harmonic IC are unlikely to be fully explained by the visual complexity factors examined here, consistent with the interpretation that our participants were sensitive to harmonic predictability during silent music reading. We are grateful to the Editor for this valuable insight, which provided us with an opportunity to enhance the robustness of our findings.

Building on Reviewer 1's observation that your model assigns high IC values to the final chords (cadences) of the 8-bar progressions, I am concerned about a significant psycholinguistic confound known as "wrap-up effects." This is the well-documented tendency for participants in self-paced reading tasks to pause at the end of a phrase to integrate meaning, regardless of the difficulty of the specific token (e.g., *Just & Carpenter, 1980*).

In Figure 1, the penultimate chord (dominant) and the final chord (tonic) are assigned IC values of 0.39 and 0.35, respectively, which are among the highest values in the sequence. If your high-IC chords are predominantly located at phrase boundaries, the correlation between IC and RT may simply reflect participants lingering at the double-barline. You must demonstrate that your results hold when controlling for chord position (specifically the final position).

Response: We thank the Editor for drawing our attention to this important psycholinguistic confound. The possibility that wrap-up effects might account for the IC-RT relationship is a legitimate concern, and we are grateful for the opportunity to address it directly.

We first examined how IC values were distributed across chord positions in our stimulus set. As with the visual complexity analyses described above, we focused on chord positions 2–8 (n = 322 chords) to maintain consistency with the data included in our linear mixed-effects models. This analysis revealed a pattern that was opposite to what would be expected if wrap-up effects were driving our results: position 8 (the final chord of each progression) had the lowest mean harmonic IC ($M = 0.32$, $SD = 0.03$) among positions 2–8, significantly lower than the average of positions 2–7 ($M = 0.34$, $SD = 0.03$; Mann-Whitney $U = 4510$, $p = .002$, $r = .18$). The moderate negative correlation between chord position and harmonic IC (Spearman rho = -.36, $p < .001$) indicates that harmonic IC tended to decrease as chord progressions unfolded, with later positions generally associated with lower harmonic IC values.

This distribution has implications for interpreting our findings. Because progression-final chords have the lowest—not highest—harmonic IC values in our stimulus set, any wrap-up-related slowing at these positions would tend to work against observing an IC effect. If participants paused at position 8 regardless of harmonic content, and these chords happened to be the most predictable, this would tend to attenuate rather than inflate the positive relationship between harmonic IC and reading times. The IC effect we observed may therefore represent a conservative estimate of the underlying relationship.

We recognize that the Editor's concern was partly prompted by Figure 1 in our original manuscript, which depicted an example progression where position 8 happened to have relatively high IC values. We acknowledge that this example was not representative of the overall distribution of harmonic IC across our stimuli. We have now replaced Figure 1 with a progression that better illustrates the typical pattern observed in our stimulus set, where harmonic IC tends to decrease toward the end of the sequence. We note that all 46 chord progressions used in this study, along with their complete harmonic IC values for each chord position, are publicly available in our Open Science Framework (OSF) repository (https://osf.io/gaz2t/), allowing independent verification of these patterns.

Nevertheless, to provide direct evidence against the possibility that wrap-up effects account for our findings, we conducted a sensitivity analysis excluding all progression-final chords (n = 4,692 observations remaining). The harmonic IC effect remained significant ($b = 1.18$, $SE = 0.35$, $t(320.93) = 3.40$, $p < .001$). This finding provides evidence against the possibility that the IC-RT relationship is an artifact of end-of-sequence processing.

These analyses are now reported in the Results section (p.45) and detailed in S1 File (pp.21–23). We hope these additions adequately address the Editor's concern.

I share the serious concerns raised by both reviewers regarding the implementation of IDyOM. You must address Reviewer 1's observation that the model appears to identify stable cadential chords as "high entropy/unpredictable". If the model assigns high information content to

structurally stable events, the validity of using these values to predict reading times is fundamentally compromised.

Also, you must clarify how a model typically used for monophonic sequences was applied to polyphonic/chordal data, as noted by Reviewer 2.

Response: We sincerely thank the Editor for emphasizing these critical concerns. We have provided detailed responses to both issues in our replies to the respective reviewers. Regarding the first concern that IDyOM assigns high harmonic IC to structurally stable cadential chords, we address this in our response to Reviewer 1 under the "2.2. Predictability" section. In brief, we clarify that IDyOM measures contextual surprisal—the conditional probability of an event given its preceding context—rather than structural importance as conceptualized in music-theoretic frameworks. This distinction may help explain why structurally central chords can still receive variable IC values depending on their local context. We elaborate on this point, including relevant empirical evidence from prior IDyOM research, in our detailed response to Reviewer 1.

Regarding the second concern—how IDyOM was applied to polyphonic/chordal data—we would like to clarify that we utilized IDyOM's harmony modeling functionality, as documented in the official IDyOM tutorial (https://github.com/mtpearce/idyom-tutorial/blob/main/tutorial-script.lisp). By specifying the *:texture :harmony* parameter, IDyOM processes vertical sonorities as unified sequential events rather than treating individual pitches as separate tokens. The target viewpoint *h-cpitch* represents each chord as the complete set of simultaneously sounding pitches, and we employed complementary source viewpoints including *pc-set* (the unordered pitch-class set present in the chord, abstracting away voicing and octave placement; e.g., a C major triad in any voicing yields the set {0, 4, 7}), *pc-set-rel-bass* (the pitch-class content expressed relative to the bass note, capturing information about chord inversion), and *root-sd* (the pitch-class of the chord root relative to the tonic of the prevailing key, representing harmonic function; e.g., 0 semitones = tonic chord, 7 semitones = dominant chord) to capture different aspects of harmonic structure. We have expanded our Methods section (pp.19–21 for melodic IC and pp.24–26 for harmonic IC analyses) to include complete technical specifications of both our harmonic and melodic IC analyses. We also provide a comprehensive explanation in our response to Reviewer 2. We hope these responses, detailed in the relevant sections below, adequately address the Editor's concerns, and we remain grateful for the guidance that has helped us strengthen the manuscript.

I agree with the reviewers that the claim that "it is still unknown whether harmonic syntax can be processed without auditory input, through visual symbols alone" is overstated. You must revise the Introduction to accurately reflect the existing state of the art (citing the literature noted by Reviewer 2, such as *Sammler et al., 2013* and *Bianco et al., 2016*), extending the field rather than claiming a "first empirical demonstration"

Response: We sincerely thank the Editor for this guidance and fully agree that our original framing was overstated. We have revised our manuscript to properly cite and discuss the important prior studies by Sammler et al. (2013) and Bianco et al. (2016). We now frame our contributions as an extension of this existing literature rather than a first demonstration of visual processing of harmonic syntax. Specifically, we clarify that while these pioneering studies

demonstrating that harmonic syntax processing may not be strictly bound to the auditory modality—showing sensitivity through visual observation of musical actions combined with motor imitation—our study explores whether similar sensitivity can emerge during the processing of symbolic musical notation alone, without any motor task component. This context is arguably more directly analogous to sight-reading, where musicians extract harmonic information from written notation rather than from observing others' actions, and parallel to word reading in language. We have tried to present this contribution with appropriate caution, acknowledging that further research would be needed to fully understand the relationship between these different forms of visual processing. Please find the revised Introduction (pp.13–14) and Discussion sections (p.52) for these changes. We remain open to any further suggestions that the Editor may have.

1.2. For the Journal Requirements:

Response:

1) PLOS ONE style requirements: We have carefully reviewed the PLOS ONE style templates and have revised our manuscript to ensure it meets all formatting requirements, including file naming conventions.
2) ORCID iD: We have added and validated the ORCID iD for the corresponding author in Editorial Manager as requested.
3) Interview transcripts: We appreciate the Editor raising the important point regarding data sharing. However, we believe there may be a misunderstanding: our study does not include any interview transcripts. The research involved behavioral tasks (sight-reading performance, self-paced reading of musical notation, and audiation test) along with a brief demographic and musical questionnaire. No interviews were conducted as part of this study. Accordingly, our data consist of (a) MIDI recordings of piano performances, (b) reading time data from the self-paced reading task, (c) scores from the audiation task, and (d) written responses to a questionnaire. None of these contain interview transcripts or sensitive identifying information beyond basic demographics, which have been de-identified. We would be happy to provide any additional clarification or documentation regarding our data if needed.
4) Citing recommended works: We have carefully reviewed the works recommended by the reviewers, specifically Sammler et al. (2013) and Bianco et al. (2016). We agree that these studies are highly relevant to our research, and we have now incorporated them into both the Introduction (pp.13–14) and Discussion sections (p.52) of the revised manuscript. We are very grateful to the Editor and reviewers for bringing these important studies to our attention.

2. Comments from Reviewer 1

The study addresses the processing of harmonic syntax through visual notation, conceptualizing such processing ability as sensitivity to the predictability of chords, and asking whether it is related to sight-reading proficiency. The study addresses harmonic processing in terms of the reading times of chords in a self-paced reading task. In statistical analyses, the reading times are modeled by computationally produced values of harmonic predictability for chords, as well as by participants' performance in a sight-reading task and audiation task. The authors could not find an effect of sight-reading or audiation skill on the reading times in the self-paced reading task, but they did find a connection between the calculated harmonic predictability of the chords and the reading times.

There are many laudable features in the manuscript. For instance, the research background is well explained, the measures of sight reading proficiency are well chosen, and the statistical analyses are appropriately carried out. The supplemental file gives a lot of useful additional information about the analyses, and the language is very good. However, I find that there are a number of interlinked problems that call the results of the study into question. The problems have to do with the authors' notion of processing harmonic syntax and how it is operationalized in the study, as well as the unusual conception of harmonic predictability that the main results rely on. I have tried to explain these problems in more detail below. Unfortunately, I think the problems run too deep into the basic study design that they cannot be very easily mended without a total reanalysis of the data, most likely using another approach to the question of harmonic predictability. Therefore, my recommendation is resubmission after a major revision.

In the following, I will first address three important problem areas, and thereafter list a number of smaller suggestions or corrections. I hope these observations could help the authors as they continue to work on their study.

Response: We are sincerely grateful to Reviewer for the thorough, thoughtful, and constructive evaluation of our manuscript. The detailed comments have been invaluable in helping us identify areas where our arguments needed strengthening and our claims required more careful qualification. We have learned a great deal from engaging with these critiques. Below, we address each of the Reviewer's concerns, organized around the three main issues raised: the methodology and interpretation of the self-paced reading task, interpretation of IDyOM predictability values, and our claims regarding visual processing of harmonic syntax.

<u>2.1. Reading Times:</u>

The self-paced reading task is central to the whole study, so it should be much better explained. First, it is unclear how exactly the stimuli were presented (did the participants see the chords sequentially at the center of the screen, or were the chords somehow successively highlighted in an entire visible score, or what?). Second, it appears that there was no specific instruction in the self-paced reading task that would make it obvious how the reading times should be interpreted. Now, the authors simply assume that longer reading times indicate "processing costs," but this seems an a priori assumption, given that the task instructions did not seem to have called for quick responses. Alternatively, longer reading times might also reflect interest, attention to

structurally central elements, or the like. Notice that such interpretations would be quite contrary to what is now simply taken for granted. In my view, the interpretation of the main results therefore lies on shaky ground: we cannot actually be sure what the reading time data is about, and thus what is being explained.

This unclarity of the main focus is somewhat masked in the abstract where it is suggested that the study addresses the research gap regarding "whether sensitivity to harmonic syntax predicts better sight-reading" (p. 2). Given the analyses conducted in this study, this is not really the gap that is addressed. In the study, the authors instead focus on questions such as whether various sight-reading metrics moderate the effect of harmonic predictability on reading times in the self-paced reading task. This is clearly a different question, and one that again direct focus to understanding the reading times in the silent-reading task which has been unconstrained by instructions that would make it easily interpretable.

Response: We thank the Reviewer for emphasizing the central importance of the self-paced reading task to our study and for identifying areas where our description and justification were insufficient. We greatly appreciate these concerns and have substantially revised the relevant sections to provide clearer explanation and stronger theoretical grounding.

For stimulus presentation, we apologize for the lack of clarity in our original description of how stimuli were presented. We have now provided a detailed account in the revised Methods section (p.29). Indeed, participants viewed chord progressions using a moving-window presentation method. At the start of each trial, only the key signature appeared on the left side of the screen. When participants pressed the spacebar, the first chord (measure 1) appeared to the right of the key signature. Each subsequent spacebar press caused the current chord to disappear and the next chord to appear at the following position to the right, so that only one chord was visible at a time while the presentation progressed spatially from left to right across the display. The key signature remained visible throughout the entire trial, allowing participants to maintain awareness of the tonal context. Reading time (RT)—the duration between successive spacebar presses—was recorded for each chord, capturing the time participants spent processing each harmonic event before choosing to advance. To further clarify this procedure, we have added a new figure (Figure 3 in the revised manuscript) illustrating the trial flow with examples that show the sequence of displays participants encountered.

The Reviewer rightly points out that our interpretation of longer reading times as reflecting processing costs represents an assumption that requires justification. We appreciate this concern and recognize that we should have made the theoretical background more explicit.

The self-paced reading paradigm has been widely used in psycholinguistic research to examine on-line language processing since the 1980s. A key assumption underlying this paradigm, sometimes called the "immediacy assumption" (Just & Carpenter, 1980), is that readers tend to process each element as it is encountered and that the time spent may reflect the cognitive demands of that processing. Research comparing self-paced reading times and eye-tracking measures has shown that word-level effects are generally similar across the two paradigms (Just et al., 1982). Additionally, information-theoretic research has found that surprisal—the negative log probability of a word given its context, analogous to our harmonic IC—tends to correlate

positively with reading times across diverse constructions (Hale, 2001; Levy, 2008; Smith & Levy, 2013).

Regarding task instructions, participants were indeed instructed to "read each chord carefully at a natural pace" and "take the time you need, but no more," ensuring they actually understood each chord before proceeding. They were also told to "avoid clicking through the chords too quickly or mechanically." These instructions, along with comprehension questions, were designed to encourage attentive reading and to ensure that participants were processing each element for comprehension rather than merely clicking through, consistent with standard practice in self-paced reading research (Jegerski, 2014; Keating & Jegerski, 2015). While the instructions did emphasize careful, engaged reading, they did not impose time pressure or instruct participants to respond as quickly as possible, allowing natural variation in processing time to emerge. We have clarified these instructions in the revised Methods section (pp.29–30).

Regarding alternative interpretations of reading time variations, we take seriously the Reviewer's thoughtful observation that longer reading times might reflect interest, attention to structurally central elements, or similar factors rather than (or in addition to) processing difficulty. We note, however, that these interpretations may not be entirely distinct from IC effects—for instance, high-IC events may inherently be more interesting or attention-grabbing precisely because they are less predictable. Nevertheless, this is an important consideration that deserves careful discussion, and we have now addressed it explicitly in the Discussion (pp.55–56).

We offer several observations that may be relevant to evaluating these alternatives, while acknowledging that behavioral data alone cannot definitively resolve questions about underlying cognitive processes. First, if longer reading times reflected positive engagement with familiar or aesthetically preferred harmonic events, we might expect trained musicians to linger on conventional progressions—yet the pattern we observed was the opposite, with less predictable chords receiving longer reading times. (Of course, less predictable events might themselves be more interesting, in which case this version of the "interest" interpretation would align with rather than contradict our findings.) Second, as noted above, our analysis of chord positions showed that final chords, which might be considered structurally salient as phrase-ending events, actually had the lowest mean IC values and did not drive the observed effect; the IC-RT relationship remained significant when excluding final-position chords entirely (for details of these IC and IC-RT analyses, please find our response to "2.2. Predictability"). Third, the relationship between surprisal and reading times has been replicated in psycholinguistic research across diverse languages and constructions (Smith & Levy, 2013; Wilcox et al., 2023), providing converging evidence for the processing-cost interpretation in linguistic contexts.

While these observations seem more consistent with the processing-cost interpretation—and while some versions of the "interest" account may not be truly distinct from IC effects, as unexpected events could inherently be more engaging—we acknowledge that we cannot entirely rule out alternative explanations with behavioral data alone. We have added this point to the Discussion (p.56), noting that future research employing converging methods—such as eye-tracking during score reading or ERP measures of harmonic expectancy (e.g., ERAN)—would help clarify the cognitive processes underlying reading time variations in musical contexts.

We hope these revisions adequately address the Reviewer's concerns about the self-paced reading task, and we are deeply grateful for the constructive feedback that prompted us to strengthen this central aspect of our study.

2.2. Predictability:

Regarding the self-paced reading task, the authors write that "The distribution of the IC values (Fig 2) confirmed that our stimuli encompassed a representative range of harmonic predictability typical of Western tonal music" (p. 21). This seems questionable. In the example chord progression (Fig. 1), all chords represent triads within the diatonic scale, and the harmonic IC values range between 0.31–0.39. According to Fig. 2, most of the chords in the stimuli indeed fell within this range. In the example progression (Fig. 1), however, the "most surprising" events are now the beginning tonic triad and the penultimate dominant triad! The authors should remember that these chords are taken as almost axiomatic points of stability in standard theories of Western tonal syntax (e.g., Schenker or GTTM). See, for instance, GTTM's time-span reductions in which the structural beginning and end of a composition (I […] V–I) are taken as the most stable events (e.g., Lerdahl & Jackendoff, 1983, p. 144).

It is indeed a bit difficult to understand why, for instance, the third last chord (I) of Fig. 1 is evaluated as more predictable as either of the two last cadential chords (V and I). It is hard to think about any usual kind of notion of harmonic predictability that this would conform with. If these are really the values produced by the Information Dynamics of Music framework, the authors should understand that such values are simply not convincing and that readers may well raise questions about the well-foundedness of the premises behind the computational procedure. At the very least, it certainly should not be the case that a "representative range of harmonic predictability typical of Western tonal music" is one in which chords that are usually taken as the most stable and predictable ones turn out to be the "least predictable" events! The authors should seriously consider using some other computational model for assessing the syntactic role of chords (e.g., Lerdahl's Tonal Pitch Space model).

The authors also easily slide from their special notion of predictability to talking about functional hierarchies. The reader may ask what grounds there are to claim that harmonic predictability, as operationalized here, has to do with "the functional, hierarchical system of rules between chords" (p. 47)? Again, the authors should really understand how questionable it is to suggest that the main functional chords in a composition as the final V and I in Fig. 1 are here claimed to be "less predictable" chords. It is highly misleading to suggest that such a strange conception represents "the functional, hierarchical system of rules between chords."

All this is also obviously linked to the interpretation of the results. Now, the authors write that there were "consistent processing costs for harmonically less predictable (high harmonic IC) chords" (p. 37). To use the above example again, the authors appear to believe the cadential dominant triad to be among the "harmonically less predictable" chords, and that the unpredictability of such a chord would lead to higher "processing costs." However, disregarding the algorithm used here, any basic understanding of tonal syntax should actually suggest such a chord to be most highly predictable in Bach-style chorale writing in the penultimate position. If so, we have reason to revisit the above criticism regarding the assumption that longer reading

times indicate processing costs: perhaps the readers might have shown more interest in such structurally important chords?

Response: We are grateful to the Reviewer for the careful examination of Figure 1 and the thoughtful questions raised about the relationship between harmonic IC values and music-theoretic notions of structural stability. This feedback has prompted us to reconsider both the choice of example and the theoretical framing of our findings, and we appreciate the opportunity to clarify these important issues.

The Reviewer raises an important point that reflects a natural and reasonable expectation: that predictability should align with structural importance as conceptualized in traditional music-theoretic frameworks such as the Generative Theory of Tonal Music (GTTM; Lerdahl & Jackendoff, 1983) or Schenkerian analysis. In these frameworks, tonic and dominant chords occupy hierarchically privileged positions and might therefore be expected to be highly "predictable" in some sense. However, as we understand it, IDyOM is designed to capture a somewhat different aspect of musical experience: conditional probability given the preceding context, expressed as Information Content (IC = $-log_2 P(event \mid context)$). This represents a measure of statistical surprisal rather than a measure of hierarchical structural importance as conceptualized in music-theoretic frameworks. We note that IDyOM captures both local sequential patterns and global stylistic regularities through its training on a musical corpus; however, its output (IC) reflects contextual predictability rather than a chord's position within a tonal hierarchy.

We believe this distinction may help clarify our findings. Corpus studies have shown that chords with roots I and V together constitute over two-thirds of all chords in major-mode passage (Moss et al., 2019)—these chords are indeed "central" in terms of overall frequency. Yet frequency alone does not determine IC; IC depends critically on the specific preceding context. Sears et al. (2018), using IDyOM to model expectations for cadential patterns in Haydn's string quartets, found that terminal events from cadential contexts (e.g., V → I progressions) received significantly lower IC estimates than non-cadential contexts featuring the same tonic harmony. This suggests that the same tonic chord might be highly predictable when approached via a cadential formula, yet relatively surprising when appearing in an unexpected sequential position.

We would suggest, therefore, that structural importance and statistical expectedness may be understood as related but separable dimensions: a chord might be structurally central yet contextually surprising, or structurally peripheral yet highly predictable given its local context.

The specific pattern the Reviewer notes—higher IC for initial tonic and penultimate dominant may be understood within this framework. Initial chords lack preceding context within the piece, so predictions rely primarily on long-term statistical knowledge acquired from the training corpus. Depending on the training corpus and the specific chord, this may result in relatively higher IC values compared to later positions where both long-term and short-term models can contribute to predicting the subsequent event. Penultimate dominant chords, while structurally expected at cadences from a music-theoretic perspective, can still vary in their contextual predictability depending on what precedes them. The same V chord will receive different IC values depending on whether it follows a conventional predominant such as IV or ii versus a less

common chromatic predominant (e.g., ♭VI)—even though both instances serve the same structural function.

Regarding Figure 1, we acknowledge that the original example progression was not representative of the typical IC distribution in our stimulus set. The example happened to show relatively high harmonic IC values at the final positions, which we recognize could be misleading. Upon closer consideration, we also recognize that the specific example we originally chose was not representative of our stimulus set. We have now replaced Figure 1 with a progression that better illustrates the pattern typically observed in our stimulus set. In our stimuli, harmonic IC values tended to decrease particularly toward the end of progressions, likely reflecting both the conventional cadential structure of our materials and the tendency for accumulating context to constrain predictions when sequences follow stylistically consistent patterns. All 46 chord progressions with their complete harmonic IC values are publicly available in our OSF repository (https://osf.io/gaz2t/), allowing independent verification of these patterns.

More importantly, when we examined IC values across all chord positions in our stimulus set, position 8 (the progression-final chord) had the lowest mean IC ($M = 0.32$, $SD = 0.03$) among positions 2–8, significantly lower than the average of positions 2–7 ($M = 0.34$, $SD = 0.03$; Mann-Whitney $U = 4510$, $p = .002$, $r = .18$). Position 1 was excluded because the linear mixed-effects models included a lag-1 reading time predictor to control for temporal autocorrelation, which necessarily removes the first chord of each progression from the behavioral analyses. By applying the same constraint to our correlation analyses, we ensured that the stimulus characteristics examined correspond directly to the data included in the confirmatory models. The correlation between chord position and IC was significantly negative (Spearman rho = -.36, $p < .001$). In our stimulus set, we observed that IC values tended to decrease with chord position. This pattern likely reflects both the specific harmonic structure of our stimuli and how statistical learning models like IDyOM operate: as more context accumulates within a sequence, the model's predictions may become more specific, and when subsequent events align with these predictions—as tends to occur in stylistically conventional progressions—this can result in lower IC for later events. Additionally, the short-term model (STM) component of IDyOM learns piece-specific patterns incrementally during processing, which could contribute to reduced surprisal for later positions when local patterns are consistent with learned regularities. We note, however, that this is not a universal property of the model—unexpected events can still receive high IC values regardless of position.

This distribution of harmonic IC across chord positions has implications for interpreting our findings in relation to the Reviewer's concern. The Reviewer raises the thoughtful possibility that longer reading times for certain chords might reflect attention to structurally central elements rather than processing costs for statistically unexpected events. We note that these interpretations may not be entirely mutually exclusive—for instance, statistically unexpected events might inherently draw more attention precisely because of their low predictability. Nevertheless, if participants were allocating more time to structurally important positions such as cadential chords regardless of predictability, we would expect elevated reading times at final positions. Because progression-final chords actually have the lowest harmonic IC values in our stimulus set, any such position-related slowing would tend to work against observing an IC effect. The positive relationship between harmonic IC and reading times that we observed may

therefore represent a conservative estimate of the underlying relationship. To provide direct evidence on this point, we conducted a sensitivity analysis excluding all progression-final chords (n = 4,692 observations remaining). The harmonic IC effect remained significant (b = 1.18, SE = 0.35, $t(320.93) = 3.40$, $p < .001$), providing evidence against the possibility that the IC-RT relationship is primarily driven by attention to structurally salient phrase-ending positions.

To illustrate the context-dependence of IC more concretely, we examined two progressions from our stimulus set that both end with authentic cadences (V^7–I/i) but differ in their preceding context. Progression A (from Schubert's "Erlkönig"; stimulus ID: prog34_schub.erlkonig.II) is in G minor and follows the progression [i – V^7/iv – iv – ♭II – ♭II – $vii^{\circ 7}$/V – V^7 – i]. This progression features chromatic elements including a Neapolitan chord (♭II) and an applied fully diminished seventh chord ($vii^{\circ 7}$/V) leading to the dominant. The IC values for chords 2–8 were 0.354, 0.347, 0.384, 0.349, 0.301, 0.314, and 0.357, respectively. Progression B (from Schubert's Impromptu Op. 142 No. 1; stimulus ID: prog36_schub.imp1) is in A♭ major and follows the progression [I – $vii^{\circ 4}{}_{2}$/ii – I – IV – $V^4{}_2$ – I^6 – V^7 – I]. This progression remains largely diatonic, with a conventional cadential pattern [I^6 – V^7 – I] at the end. The IC values for chords 2–8 were 0.345, 0.352, 0.332, 0.329, 0.352, 0.297, and 0.289, respectively.

In both progressions, the V^7 chord appears at position 7, serving the same structural function as the penultimate dominant in an authentic cadence. However, the IC values appear to differ: 0.314 in Progression A versus 0.297 in Progression B. We suggest this difference may be attributed to the preceding harmonic context. In Progression A, the dominant is approached through chromatic voice leading from $vii^{\circ 7}$/V, following a passage that includes the Neapolitan chord—a relatively marked harmonic event. In Progression B, the dominant is approached from I^6, completing a conventional cadential formula [IV – $V^4{}_2$ – I^6 – V^7 – I] that is highly typical in common-practice tonal music.

The IC values for the final tonic chord (position 8) also differed between the two progressions: 0.357 in Progression A versus 0.289 in Progression B. It would be worth noting that the change in IC from the dominant to the tonic seemed to differ in direction: in Progression A, IC increased from 0.314 (V^7) to 0.357 (i), whereas in Progression B, IC slightly decreased from 0.297 (V^7) to 0.289 (I). This pattern might suggest that in the diatonic context of Progression B, the tonic resolution was highly predictable once the dominant appeared—consistent with the conventional cadential formula. In contrast, the preceding chromatic events in Progression A (including the Neapolitan and diminished seventh chords) may have introduced sufficient harmonic ambiguity that even the final tonic resolution remained relatively less predictable. Although both progressions end with the same structural cadence [V^7 – I/i], their IC values appear to differ because of their local contexts. We hope this example may help illustrate how IC captures contextual predictability rather than chord identity or structural function per se, though we acknowledge that further investigation would be needed to fully characterize this relationship.

Progression A (from Schubert's "Erlkönig"; stimulus ID: prog34_schub.erlkonig.II)

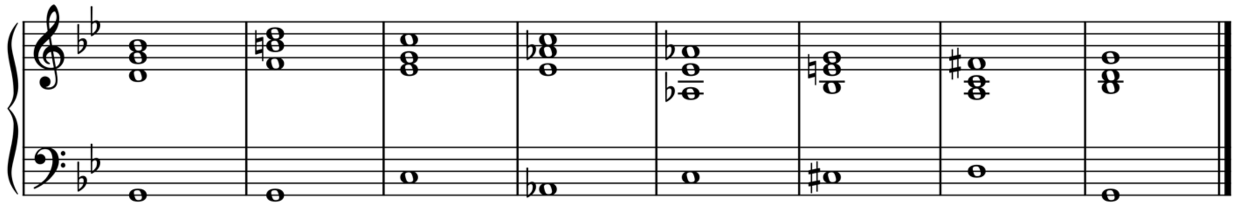

Progression B (from Schubert's Impromptu Op. 142 No. 1; stimulus ID: prog36_schub.imp1)

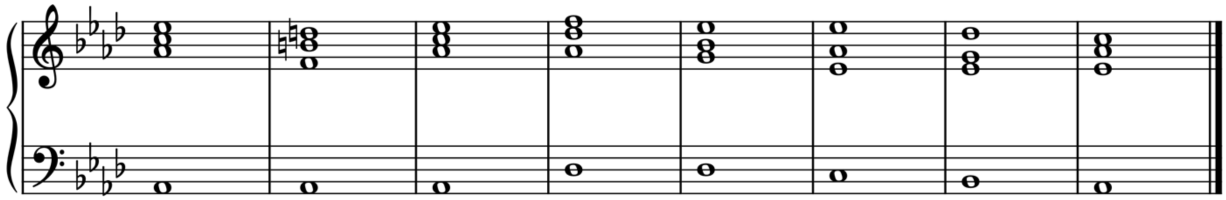

We chose IDyOM for our study because it provides a quantitative, information-theoretic measure of harmonic expectation grounded in statistical learning principles. IDyOM has been extensively validated in the melodic domain, where model-derived estimates correlate with behavioral and neural measures of expectation (e.g., Di Liberto et al., 2020; Hansen & Pearce, 2014; Omigie et al., 2013; Pearce, 2018; Quiroga-Martinez et al., 2020). Additionally, researchers have begun applying the framework to harmonic sequences, with results linking model estimates to listeners' affective responses and neural activity (Cheung et al., 2019; Sears et al., 2018). The underlying principle—that expectations arise from internalized statistical regularities learned through exposure—is domain-general and may therefore extend to visual processing of musical notation, which is the hypothesis we sought to test.

Regarding the Reviewer's suggestion to consider Lerdahl's Tonal Pitch Space (TPS) model as an alternative, we appreciate the opportunity to clarify our rationale. TPS quantifies perceived distances between chords within a hierarchical tonal framework, capturing structural relatedness rather than sequential probability. While TPS would be well-suited for examining how structural distance affects processing, our study focuses on how probabilistic expectations may influence reading times—a question that, we believe, requires a model like IDyOM that generates predictions based on learned sequential regularities. These approaches thus address complementary but distinct research questions. We hope this clarification addresses the Reviewer's question regarding our choice of model.

We would also like to note that our stimulus set was drawn from a range of common-practice tonal music, including works by Bach, Mozart, Haydn, Beethoven, Schubert, Schumann, Brahms, Chopin, and Tchaikovsky, among others. This diversity of repertoire may help ensure that the harmonic IC patterns we observed reflect general properties of Western tonal harmony rather than idiosyncrasies of a single composer's style.

We have also expanded the Discussion (pp.54–55) to more explicitly acknowledge the distinction between statistical predictability and structural importance, and we hope this clarification addresses the Reviewer's concern. We are deeply grateful for the opportunity to sharpen our theoretical framing.

2.3. Processing of Harmonic Syntax:

The authors claim that "it is still unknown whether harmonic syntax can be processed without auditory input, through visual symbols alone" (p. 11). Let me just note first that, at face value, this may sound like a rather absurd statement, given that generations of music students have been taught to do exercises in harmonic analysis by silently reading musical notation! That surely counts as processing harmonic syntax. Therefore, I would strongly recommend that the authors do not use these kinds of generalizing statements when talking about sensitivity to chord predictability. The conclusion "This study provides the first empirical demonstration that harmonic syntax can be processed visually without auditory input" (p. 52) thus appears misleading, and may seem like an attempt to artificially inflate the value of the study.

The authors do explain what they mean with processing harmonic syntax (even though this could be done earlier), and it helps a bit, but not entirely. Visually processing harmonic syntax is explained to mean "the ability to extract harmonic relationships from musical notation without auditory input, measured through sensitivity to harmonic predictability," "through the degree to which reading times increase according to the harmonic predictability of the chords in the self-paced reading task" (pp. 13–14). In other words, using more silent-reading time for "less predictable chords" than "predictable" ones "indicates more efficient extraction and utilization of syntactic information from visual notation" (p. 14). However, assuming that reading times indeed reflect processing costs, we may now ask why would relative difficulties in reading less predictable chords indicate "more efficient extraction and utilization of syntactic information"? Given that all of the chords seem to have been diatonic triads, why couldn't an efficient extraction be conversely reflected by smaller differences in reading time between the different chords? Again, it seems that the authors are simply assuming something.

Note that if some of chords evaluated by the algorithm as "less predictable" are actually among the most common tonal chords (see above), the readers' use of time for reading the different chords could also then be interpreted as an indication of processing harmonic syntax. However, in that case the interpretation from "predictability" would need to be revised, e.g., by suggesting that the participants have used most reading time for the structurally central elements. There seems to be nothing in the study design that would rule out such a contrary interpretation. The participants were experienced musicians: why should their relative use of time between tonal triads primarily be interpreted in terms of relative surprisingness, instead of assuming that their reading process can also reflect something of the relative functional hierarchies in the tonal system?

Even given the restricted definition of "processing harmonic syntax" in terms of sensitivity to chord predictability, the authors' broader claims tend to slide into more general conclusions regarding "a more general cognitive principle for processing structured sequential information" (p. 44). However, it should be remembered that the data has to do with reading times for chords. The same conclusions might have been reached by a reductio ad absurdum version of the study, where the stimulus chords would include both very conventional chords (e.g., {C, E, G}) and less conventional and syntactically unclear ones (e.g., {C, Db,Gb}). It could be expected that silent reading times for the latter would often be longer than for the former, and that some similar differences could be obtained in a parallel listening task. I suspect, then, that it would be rather

easy to get such a result with stimuli that would also more convingly align with standard notions of chord predictability.

But the question remains if there is anything interesting about such a result (whether in the present study or in my above hypothetical example)? How do such results about reading times help us draw the conclusion “that music and language not only share neural resources for syntactic processing but that these resources operate across sensory modalities” (p. 44)? How should the silent-reading times on the scale of seconds (that may or may not reflect the subjective difficulty of the stimuli, their predictability, or the significance attributed to them) be related to involuntary brain responses in listening studies that appear on a much smaller time scale? It appears to me that the authors are presenting results that are not very surprising, but also strongly overstating their importance for general conclusions about music cognition. In the authors’ approach, the notion that something “can be processed” (p. 46) in a given modality comes very cheap: it does not seem to require much more that we can show that some syntactic elements take more time to tackle than others—whatever the task is and whatever the potential reasons for using different amounts of time.

Response: We are grateful to the Reviewer for raising important concerns about our claims regarding visual processing of harmonic syntax. This feedback has helped us recognize that several of our formulations were imprecise or overstated, and we have made revisions throughout the manuscript.

The Reviewer points out that our statement that “it is still unknown whether harmonic syntax can be processed without auditory input” may seem problematic given that generations of music students have performed harmonic analysis by silently reading notation. We appreciate this observation and recognize that our original phrasing was indeed imprecise. We certainly did not intend to suggest that musicians cannot extract harmonic information from notation—clearly they can and have done so for generations. Our wording did not adequately specify what we meant by “processing” in this empirical context, and we are grateful for the opportunity to clarify.

What we intended to examine is not whether musicians can extract harmonic information from notation per se, but whether this extraction exhibits graded sensitivity to the statistical predictability of harmonic events as measured by information-theoretic models. Our operationalization of “processing harmonic syntax” is specific: we assess whether reading times vary systematically as a function of harmonic IC—that is, whether readers show what appear to be processing costs that track probabilistic expectations. This follows the approach used in psycholinguistic self-paced reading research, where reading time modulation by predictability is taken as one form of evidence for on-line syntactic processing.

We have revised all relevant passages to make this distinction explicit. For example, in the Introduction (p.11), we now state that “…while musicians may engage in harmonic analysis from notation alone (e.g., in a music theory classroom examining a musical score by eye), it is yet uncertain whether such visual processing exhibits similar sensitivity to harmonic predictability observed in auditory paradigms…” In the Conclusions (p.63), we have replaced “first empirical demonstration that harmonic syntax can be processed visually” with language

specifying what our study actually provides evidence for: “This study provides evidence suggesting that sensitivity to harmonic predictability may extend to the visual modality: even without auditory input, participants showed longer reading times for less predictable chords, suggesting that expectation-based harmonic processing may occur through symbolic notation alone. This finding highlights the importance of visual processing in musical syntax research and opens new questions about modality-independent representations: our results suggest that harmonic-syntactic knowledge may be accessible not only through auditory perception and visuomotor simulation, but also through the processing of abstract visual representations in written notation.…”

The Reviewer also questions our characterization that larger reading time differences indicate “more efficient extraction and utilization of syntactic information.” Upon reflection, we agree that this was a misleading formulation. Larger reading time differences do not indicate greater efficiency; rather, they may indicate greater sensitivity—that is, the reader’s processing appears to be modulated by the statistical properties of the harmonic sequence. A reader whose RTs vary with harmonic IC is demonstrating that their cognitive system appears to be tracking harmonic predictability; we did not intend to make claims about how sophisticated or efficient that processing is. We have revised this passage accordingly (p.15), removing efficiency claims and focusing instead on sensitivity as potential evidence that harmonic structure is being processed.

The Reviewer also expresses concern that we may be overstating the importance of our findings for general conclusions about music cognition, and questions whether our behavioral data support conclusions about shared neural resources across music and language or about syntactic processing operating across sensory modalities. We appreciate this concern and agree that our data alone cannot establish such neural claims.

We have revised the Discussion (p.53) to present theoretical frameworks like the Shared Syntactic Integration Resource Hypothesis (SSIRH) as contexts that our findings may be consistent with, rather than claiming that our data demonstrate or extend these frameworks. We now state: “These findings appear consistent with the Shared Syntactic Integration Resource Hypothesis (SSIRH) proposed by Patel [102], which posits shared neural resources for syntactic processing across music and language. Our behavioral evidence raises the possibility that such shared resources, if they exist, might operate across sensory modalities as well, though direct neuroimaging evidence would be needed to evaluate this hypothesis more rigorously.”

We have also added explicit acknowledgment in the “Limitations and future directions” section in the Discussion (pp.62–63) that the interpretation of reading times in musical contexts may involve complexities not fully addressed in the present study, and that converging evidence from other methodologies would strengthen conclusions about the nature of visual harmonic syntax processing.

The Reviewer notes that the Abstract suggests our study addresses whether “sensitivity to harmonic syntax predicts better sight-reading,” but that our actual analyses focus on whether sight-reading metrics moderate the effect of harmonic predictability on reading times. We appreciate this observation and have revised the Abstract to more accurately reflect what our

study examines. We now frame the research gap as concerning “how individual differences in sensitivity to harmonic syntax may relate to sight-reading proficiency.”

Regarding our findings, the Reviewer suggests that our results may not be very surprising and that we may be overstating their importance. We respect this perspective and have worked to temper our claims throughout the manuscript. In response to feedback from another reviewer, we have also incorporated prior work demonstrating visual processing of harmonic syntax through action observation (Bianco et al., 2016; Sammler et al., 2013), and we no longer claim our study to be the first demonstration of visual harmonic processing. Rather, we believe our study offers a novel contribution by demonstrating that sensitivity to harmonic predictability can emerge during the reading of symbolic notation alone—without motor imitation or action observation—a context that is arguably more directly relevant to the initial stages of sight-reading, and parallel to word reading in language. We have tried to present this contribution with appropriate caution, focusing on what our data can reasonably support while remaining open to alternative interpretations.

We are deeply grateful to the Reviewer for the constructive and thought-provoking feedback, which has substantially improved the clarity, precision, and appropriate qualification of our claims.

2.4. Minor Comments:

P. 27: In the formula for CV, should the phrase over the line be “Standard deviation of absolute timing deviations”? Notice that the absence of the word “absolute” may be a bit confusing: “abolute deviations” are mentioned in the denominator, but otherwise, this has not been explained, and the reader still has in mind the beat-related deviations from the previous formula. It might be useful to define “timing deviation” outside of the bnMAD formula so that it can also be understood for CV.

Response: We sincerely thank the Reviewer for this careful observation. The Reviewer is absolutely correct that the absence of “absolute” in the numerator of the CV formula was confusing, especially given that “absolute deviations” appears in the denominator. We have made two revisions to address this issue. First, we now define “timing deviation” separately before introducing the rhythm metrics, clarifying that it refers to the difference between performed and expected onset times. Second, we have revised the CV formula to read “Standard deviation of absolute timing deviations / Mean of absolute timing deviations,” ensuring consistency between the numerator and denominator. We hope these changes make the formula clearer for readers. Please find pp.32–33 in the revised manuscript for these changes.

P. 31: If I understand correctly from the paired comparisons using the Wilcoxon signed-rank test, the authors must have first calculated mean participant values for each of the accuracy metrics. This does not seem quite clear now. For instance, the formula for Pitch Error Rate mentions the number of total notes in a score (p. 27), but there were three scores in both of the (tonal and atonal) conditions (p. 17). Please make it clear if means were taken first across the three scores before the Wilcoxon signed-rank tests.

Response: We thank the Reviewer for requesting this clarification. The Reviewer's understanding is correct: because each participant performed three pieces per condition, we first calculated each participant's mean score across the three pieces within each condition (tonal and atonal), yielding one aggregated value per participant per condition. These participant-level means were then submitted to the Wilcoxon signed-rank tests. We apologize that this was not clearly stated in the original manuscript. We have now added an explicit description of this aggregation procedure in the Results section (p.37).

P. 36: I think I understand the basic idea, but I think it would be still good to explain in some more detail how you "created lag-1 RT predictors." What does it exactly mean? Could you give an example of the values of such a variable?

Response: We appreciate the Reviewer's suggestion to provide more detail on the lag-1 RT predictor. We have expanded this section to include: 1) a clearer explanation of what temporal autocorrelation means in the context of self-paced reading data and why it poses a problem for regression models, and 2) a concrete numerical example illustrating how the lag-1 RT variable is constructed. To directly address the Reviewer's question: for each chord at position *n*, the lag-1 RT variable contains the log-transformed reading time of the immediately preceding chord at position *n*-1. For example, if a participant's log-transformed reading times for chords 1 through 4 in a progression were 3.30, 3.54, 3.45, and 3.62, then the corresponding lag-1 RT values for chords 2, 3, and 4 would be 3.30, 3.54, and 3.45, respectively. Because chord 1 has no preceding observation, it is excluded from the analysis. By including this lag-1 RT as a predictor, the model accounts for the tendency of consecutive reading times to be correlated, thereby addressing the autocorrelation issue. We hope this additional detail, now included in the revised manuscript (p.35), makes the analytical approach more transparent for readers.

Pp. 37–38: It should be more clearly stated before and in Table 3 that what is predicted here are reading times.

Response: We thank the Reviewer for pointing out this ambiguity. We agree that it should be clearer that reading times are the dependent variable in all models. We have made two changes (p.46): 1) we added a sentence before Table 3 stating "In all models reported below, the dependent variable is log-transformed reading time," and 2) we revised the Table 3 title to explicitly mention "Predicting Log-Transformed Reading Times." We hope these changes improve clarity.

P. 42/line 792: "These results fail to provide evidence that audiation ability neither enhanced nor compensated…" Because of "fail," this becomes a double negation which is clearly not meant here.

Response: We thank the Reviewer for catching this grammatical error. The double negation was unintended. We have revised the sentence to read: "These results do not provide evidence that audiation ability enhanced or compensated for the relationship between sight-reading proficiency and harmonic syntax processing in our sample." Please find p.50 in the revised manuscript.

3. Comments from Reviewer 2

While the manuscript presents a well-designed and carefully executed study, several issues require clarification and revision.

Response: We sincerely thank the Reviewer for the thoughtful and constructive feedback. The points raised have helped us recognize where our methodological descriptions needed greater clarity, and we are grateful for the opportunity to provide a more thorough explanation. Below, we address each concern in detail.

First, I disagree with the authors' statement of the knowledge gap. The manuscript claims that "it is still unknown whether harmonic syntax can be processed without auditory input, through visual symbols alone" (line 189). However, prior work has already demonstrated that musical syntax can be processed in the absence of auditory input using purely visual stimuli (e.g., Sammler et al., 2013, Cortex; Bianco et al., 2016, NeuroImage). This body of literature should be acknowledged and discussed. Moreover, the authors should clarify the specific contribution of the present study beyond these existing findings.

Response: We are deeply grateful to the Reviewer for raising this important point and for directing us to the work by Sammler et al. (2013) and Bianco et al. (2016). We fully acknowledge that our original statement of the knowledge gap was not sufficiently nuanced. We have now carefully reviewed these studies and revised our manuscript to properly acknowledge their contributions. Both studies elegantly demonstrated that pianists watching silent depictions of hands playing chord progressions—videos in Sammler et al. and photographs in Bianco et al.—exhibited neural signatures sensitive to harmonic violations, providing evidence suggesting that harmonic syntax processing may not be strictly confined to the auditory modality but can also operate through the visuomotor system.

In revising the manuscript, we have also taken the opportunity to more carefully articulate what we believe may be the specific contribution of our study. We would like to respectfully note a distinction that we hope may be relevant: the studies by Sammler et al. and Bianco et al. employed visual depictions of musical actions—videos or photographs of hands playing chords on a piano—and required participants to motorically imitate the observed movements. Their findings thus speak to harmonic syntax processing within the visuomotor or "embodied" action system, which Sammler et al. (2013, p.1336) insightfully termed "a grammar of musical action." Our study, in contrast, uses symbolic musical notation as the visual stimulus, with no motor task involved. Participants simply read chord progressions presented in standard musical notation without any imitation or performance requirements. We believe this paradigm may capture a somewhat different aspect of visual processing—one that is more abstract and potentially more analogous to the initial stages of sight-reading, where musicians extract harmonic relationships from written symbols before motor planning occurs, and parallel to word reading in language.

We do not wish to overstate this distinction, as it is certainly possible that similar or overlapping mechanisms underlie both types of processing. However, we believe that examining the sensitivity to harmonic syntax in a notation-reading context offers a novel, complementary

perspective to the action-observation paradigm, potentially contributing to a more complete understanding of how harmonic representations can be accessed through different visual formats.

We have revised the Abstract and Introduction (pp.13–14) to acknowledge the prior literature and clarify our intended contribution, and we have added discussion of these studies in the Discussion section (p.52) as well. We are sincerely grateful for this feedback, which has helped us situate our work more accurately within the existing literature. We remain open to any further suggestions the Reviewer may have.

Second, the application of the monophonic IDyOM model to harmonic material is unclear. IDyOM operates on sequences of the pitches or intervals of individual notes as tokens in an n-gram framework. It is therefore not obvious how information content was computed for homophonic and/or polyphonic materials, including the ABRSM sight-reading pieces and the four-part chords used in the self-paced reading task. For example, was only a single voice (e.g., the highest pitch) analyzed and subsequently interpreted as harmonic information content? The procedure requires explicit clarification to ensure that the reported measures are conceptually and methodologically valid.

Response: We sincerely thank the Reviewer for raising this important methodological question, and we apologize that our original description lacked sufficient detail. We would like to clarify that our study employed two distinct IDyOM analyses for different purposes, each with an approach suited to its specific goal. We describe each analysis in turn and have substantially expanded the Methods section of the revised manuscript (pp.19–21) to include complete technical specifications.

For the 46 chord progressions used in the self-paced reading task, we utilized IDyOM's harmony modeling functionality. As documented in the official IDyOM tutorial (https://github.com/mtpearce/idyom-tutorial/blob/main/tutorial-script.lisp), IDyOM can process harmonic sequences by representing vertical sonorities as unified sequential events rather than treating individual pitches as separate tokens. This is achieved by specifying the *:texture :harmony* parameter when loading musical data and running the model.

In this configuration, the target viewpoint *h-cpitch* represents each chord as the complete set of simultaneously sounding pitches. The IDyOM tutorial demonstrates the use of several harmonic viewpoints that can serve as source viewpoints for predicting *h-cpitch*. Following the tutorial examples, we employed three complementary source viewpoints: *pc-set* (the unordered collection of pitch classes present in the chord, abstracting away octave placement and voicing details), *pc-set-rel-bass* (the pitch-class content expressed relative to the bass note, capturing information about chord inversion), and *root-sd* (the pitch-class of the chord root relative to the tonic of the prevailing key, representing harmonic function). Each of these viewpoints captures a distinct aspect of harmonic structure. IDyOM combines their predictions by computing a weighted geometric mean, where the weight assigned to each viewpoint is inversely related to its entropy—that is, viewpoints generating more certain (lower-entropy) predictions contribute more strongly to the final estimate (Pearce, 2005).

We should note that IDyOM's automatic viewpoint selection procedure does not currently appear to support the harmonic texture mode; therefore, we selected these viewpoints based on the examples provided in the official tutorial documentation.

For the sight-reading materials, we conducted a separate melodic analysis with a distinct purpose from the harmonic analysis described above. Although our sight-reading pieces were written for both hands and thus contain textures that could be analyzed harmonically, the goal of this particular analysis was to objectively verify that our atonal pieces exhibited lower predictability than the tonal pieces. Since this analysis served as a manipulation check for stimulus validation—confirming that the tonal and atonal conditions differed as intended—rather than as a predictor variable in our main statistical models of reading time, melodic IC was considered sufficient and appropriate for this purpose.

For this melodic analysis, we employed IDyOM's automatic viewpoint selection procedure rather than manually specifying source viewpoints. As described in the IDyOM documentation and tutorial, this data-driven approach uses a hill-climbing algorithm to identify the combination of source viewpoints that minimizes the mean IC for the target viewpoint, which may help reduce potential researcher bias in viewpoint specification. In our analysis, the target viewpoint was *cpitch* (chromatic pitch), and the automatic selection procedure identified the following viewpoint system for our sight-reading pieces: *cpintfref* (chromatic pitch interval from the referent, representing scale degree), *cpint* (chromatic pitch interval from the previous note), *(cpitch cpintfref)* (a linked viewpoint combining absolute pitch with scale degree), and *(cpint contour)* (a linked viewpoint combining pitch interval with melodic contour direction).

Both harmonic and melodic analyses employed the *both*+ model configuration, which is the default setting in IDyOM. As described in the IDyOM documentation and Pearce (2005, 2018), this configuration combines a long-term model (LTM) that can be pretrained on external corpora with a short-term model (STM) that learns incrementally within each sequence starting from an empty state. The "+" indicates that the LTM also receives additional incremental updates during testing, which is intended to simulate how listeners may continuously refine their expectations based on incoming musical information within a piece.

For pretraining, both analyses used the Bach 371 Chorales (available from the CCARH Kern Scores collection) and the Kostka-Payne (KP) corpus (comprising 46 excerpts from common-practice repertoire, Temperley, 2009). These corpora were selected to provide the model with exposure to Western tonal conventions that might approximate the musical knowledge experienced musicians would have acquired through their training.

We have also uploaded the complete raw IDyOM output files to our OSF repository (https://osf.io/gaz2t/) so that readers can examine the full model predictions for each stimulus item.

Third, the training procedure for IDyOM raises concerns about overfitting. Because IDyOM already incorporates local context via its short-term viewpoint, additional training on the test stimuli themselves appears unnecessary and potentially problematic. If the intention was to fine-tune the model to stylistic properties, training on separate materials drawn from the same corpus

would be more appropriate. This concern is particularly relevant given the apparent stylistic similarity between the Bach corpus and the test materials.

Response: We greatly appreciate the Reviewer's careful attention to potential overfitting concerns, and we would like to clarify that our cross-validation procedures were designed to address this issue.

For the harmonic IC analysis of our 46 chord progressions, we employed leave-one-out cross-validation by setting the *k* parameter to 46. Under this procedure, when computing IC values for any given chord progression, that specific progression was excluded from the LTM training data. The LTM was trained only on the remaining 45 progressions together with the external pretraining corpora (Bach chorales and KP corpus). This approach is intended to help ensure that the LTM's predictions for each test progression reflect more generalized harmonic knowledge rather than specific features of that particular test item.

We applied the same cross-validation principle to our melodic IC analysis. For the six sight-reading pieces, we employed leave-one-out cross-validation by setting *k* to 6. When computing IC values for any given piece, that piece was excluded from the LTM training data, with the model trained only on the remaining five pieces together with the external pretraining corpora.

To clarify the intended function of each model component in the *both+* configuration as described in the IDyOM documentation: The LTM is designed to capture statistical regularities learned from the training corpora, representing knowledge accumulated from prior musical exposure. Through cross-validation, the LTM does not see the specific test item when generating predictions for that item. The STM begins in an empty state at the start of each piece and learns incrementally as the sequence unfolds, capturing local patterns such as recurring motifs within a single piece. The incremental LTM updating (the "+" in *both+*) allows the LTM to receive additional updates from the current sequence during testing, simulating a listener who continuously refines long-term expectations based on the unfolding stimulus (Quiroga-Martinez et al., 2020). Importantly, these updates occur only after each prediction is made—that is, the model first generates a prediction for each event based on the preceding context, and then incorporates that event into its representations. This causal structure prevents backward information leakage and is designed to simulate real-time expectation refinement rather than to optimize predictions for a known test set.

We acknowledge the Reviewer's concern about potential redundancy between the STM and incremental LTM updating, as both involve learning from the test sequence. However, these components are designed to capture regularities at different timescales: the STM captures short-range, piece-specific patterns, while the incremental LTM updates allow gradual refinement of longer-range expectations based on the accumulating context. This combination of long-term and short-term learning, together with cross-validation, follows standard approaches in the IDyOM literature (e.g., Omigie et al., 2013; Pearce, 2018; Quiroga-Martinez et al., 2020).

We acknowledge that our original manuscript did not make the cross-validation procedures sufficiently clear, and we have now added explicit details about the *k* parameter settings and their rationale to the Methods section of the revised manuscript (pp.21, 26).

Finally, I understand that PLOS One requires data availability for all publications. I believe the (minimal) raw data should already be available for review as well. Otherwise, how would reviewers assess the scientific rigor and integrity of the manuscript? Please include the data (or a link to the data) in the revision.

Response: We thank the Reviewer for this reminder and sincerely apologize for any confusion. The complete dataset was uploaded to our OSF repository prior to initial submission; however, we recognize that the repository link was provided only through the journal's submission portal and was not explicitly stated within the manuscript text itself, which may have made it difficult for reviewers to locate.

We have now added the OSF repository URL (https://osf.io/gaz2t/) directly to the relevant sections in the revised manuscript. Additionally, in response to this helpful feedback, we have supplemented the repository for this project with the complete raw IDyOM output files, which provide the detailed model predictions—including probability distributions, entropy values, information content for each event, and model order information—for every chord in the self-paced reading stimuli and every note in the sight-reading pieces. The previously uploaded materials already included the stimuli for the self-paced reading task, IC values for both sight-reading and self-paced reading materials, and behavioral (performance accuracy, reading time, and NESI scores) and questionnaire data. The have also uploaded our analysis code in the repository for reproducibility.

I would be happy to review a revised version of the manuscript that addresses these points.

Response: We sincerely thank Reviewer for the careful and constructive review. The feedback has been invaluable in improving the clarity and transparency of our manuscript, and we hope our revisions adequately address the concerns raised.

References

Bianco, R., Novembre, G., Keller, P. E., Kim, S. G., Scharf, F., Friederici, A. D., Villringer, A., & Sammler, D. (2016). Neural networks for harmonic structure in music perception and action. *NeuroImage, 142*, 454–464. https://doi.org/10.1016/j.neuroimage.2016.08.025

Cheung, V. K. M., Harrison, P. M. C., Meyer, L., Pearce, M. T., Haynes, J. D., & Koelsch, S. (2019). Uncertainty and Surprise Jointly Predict Musical Pleasure and Amygdala, Hippocampus, and Auditory Cortex Activity. *Current Biology: CB, 29*(23), 4084–4092.e4. https://doi.org/10.1016/j.cub.2019.09.067

Di Liberto, G. M., Pelofi, C., Bianco, R., Patel, P., Mehta, A. D., Herrero, J. L., de Cheveigné, A., Shamma, S., & Mesgarani, N. (2020). Cortical encoding of melodic expectations in human temporal cortex. *eLife, 9*, e51784. https://doi.org/10.7554/eLife.51784

Hale, J. (2001). A probabilistic Earley parser as a psycholinguistic model. In *Proceedings of the second meeting of the North American Chapter of the Association for Computational Linguistics on Language technologies*, 1–8. https://doi.org/10.3115/1073336.1073357

Hansen, N. C., & Pearce, M. T. (2014). Predictive uncertainty in auditory sequence processing. *Frontiers in Psychology, 5*, Article 1052. https://doi.org/10.3389/fpsyg.2014.01052

Jegerski, J. (2014). Self-paced reading. In J. Jegerski & B. VanPatten (Eds.), *Research methods in second language psycholinguistics* (pp. 20–49). Routledge.

Just, M. A., & Carpenter, P. A. (1980). A theory of reading: From eye fixations to comprehension. *Psychological Review, 87*(4), 329–354. https://doi.org/10.1037/0033-295X.87.4.329

Just, M. A., Carpenter, P. A., & Woolley, J. D. (1982). Paradigms and processes in reading comprehension. *Journal of Experimental Psychology: General, 111*(2), 228–238. https://doi.org/10.1037/0096-3445.111.2.228

Keating, G. D., & Jegerski, J. (2015). Experimental designs in sentence processing research: A methodological review and user's guide. *Studies in Second Language Acquisition, 37*(1), 1–32. https://doi.org/10.1017/S0272263114000187

Lerdahl, F., & Jackendoff, R. (1983). *A generative theory of tonal music*. MIT Press.

Levy, R. (2008). Expectation-based syntactic comprehension. *Cognition, 106*(3), 1126–1177. https://doi.org/10.1016/j.cognition.2007.05.006

Moss, F. C., Neuwirth, M., Harasim, D., & Rohrmeier, M. (2019). Statistical characteristics of tonal harmony: A corpus study of Beethoven's string quartets. *PLOS ONE, 14*(6), e0217242. https://doi.org/10.1371/journal.pone.0217242

Omigie, D., Pearce, M. T., Williamson, V. J., & Stewart, L. (2013). Electrophysiological correlates of melodic processing in congenital amusia. *Neuropsychologia, 51*(9), 1749–1762. https://doi.org/10.1016/j.neuropsychologia.2013.05.010

Pearce, M. T. (2005). *The construction and evaluation of statistical models of melodic structure in music perception and composition* (Doctoral dissertation, City University London).

Pearce, M. T. (2018). Statistical learning and probabilistic prediction in music cognition: Mechanisms of stylistic enculturation. *Annals of the New York Academy of Sciences, 1423*(1), 378–395. https://doi.org/10.1111/nyas.13654

Quiroga-Martinez, D. R., Hansen, N. C., Højlund, A., Pearce, M., Brattico, E., & Vuust, P. (2020). Decomposing neural responses to melodic surprise in musicians and non-musicians: Evidence for a hierarchy of predictions in the auditory system. *NeuroImage, 215*, 116816. https://doi.org/10.1016/j.neuroimage.2020.116816

Sammler, D., Novembre, G., Koelsch, S., & Keller, P. E. (2013). Syntax in a pianist's hand: ERP signatures of "embodied" syntax processing in music. *Cortex: A Journal Devoted to the Study of the Nervous System and Behavior, 49*(5), 1325–1339. https://doi.org/10.1016/j.cortex.2012.06.007

Sears, D. R. W., Pearce, M. T., Caplin, W. E., & McAdams, S. (2018). Simulating melodic and harmonic expectations for tonal cadences using probabilistic models. *Journal of New Music Research, 47*(1), 29–52. https://doi.org/10.1080/09298215.2017.1367010

Smith, N. J., & Levy, R. (2013). The effect of word predictability on reading time is logarithmic. *Cognition, 128*(3), 302–319. https://doi.org/10.1016/j.cognition.2013.02.013

Temperley, D. (2009). *A statistical analysis of tonal harmony*. http://davidtemperley.com/kp-stats/

Wilcox, E. G., Pimentel, T., Meister, C., Cotterell, R., & Levy, R. P. (2023). Testing the predictions of surprisal theory in 11 languages. *Transactions of the Association for Computational Linguistics, 11*, 1451–1470. https://doi.org/10.1162/tacl_a_00612
